# Expanding the clinical and mutational spectrum of hereditary spastic paraplegia type 4 in a cohort of patients from central China

**DOI:** 10.3389/fgene.2026.1800987

**Published:** 2026-03-30

**Authors:** Jun Fu, Jia Song, Gang Li, Mi Pang, Jiewen Zhang, Mingming Ma

**Affiliations:** Department of Neurology, Henan Provincial People’s Hospital, Zhengzhou, China

**Keywords:** complicated phenotype, hereditary spastic paraplegia, hyposmia, novel variant, SPAST, SPG4

## Abstract

**Background:**

Mutations in the *SPAST* gene cause autosomal dominant hereditary spastic paraplegia (HSP) type 4 (SPG4), which is the most common type of HSP with variable frequencies in different ethnic backgrounds. The clinical and genetic characteristics of SPG4 in Central China have not been well documented.

**Methods:**

We screened for *SPAST* variants by whole exome sequencing in a cohort of 63 unrelated families with HSP from Central China. The clinical manifestations were evaluated.

**Results:**

21 variants of *SPAST* were identified in 21 index patients with a frequency of 33.3% (21/63). Seven novel variants were identified, including one missense variant (p.S399W), five frameshift variants (p.Q170Vfs*2, p.S527Vfs*3, p.I605Vfs*17, p.I605Nfs*26, and p.V443Afs*2), and one splicing variant (c.871-1G>A). We also detected four previously reported exon deletions of *SPAST*. The mean age of disease onset was 34.0 years. Anticipation and variability of disease severity were observed in some autosomal dominant families. Two patients exhibited a complicated phenotype, one of whom presented with hyposmia, which had never been previously reported with SPG4.

**Conclusion:**

SPG4 is the most common type of HSP in our cohort. Complicated phenotype, although rare, can also be observed in SPG4 patients. The hyposmia might be a new phenotype associated with SPG4. The *SPAST* rearrangement is common and should be considered during genetic analysis. The novel *SPAST* variants identified in this study expand the mutational spectrum.

## 1 Introduction

Hereditary spastic paraplegia (HSP) comprises a group of clinically and genetically heterogeneous neurodegenerative disorders (Erfanian Omidvar et al., 2021). According to the clinical presentation, HSP is classified as pure form characterized by isolated progressive lower limb weakness and spasticity, or complicated form with additional neurological and extra-neurological manifestations, such as cognitive impairment, cerebellar ataxia, epilepsy, visual disturbance, and peripheral neuropathy (Cao et al., 2024; Harding, 1993). The modes of inheritance include autosomal dominant (AD), autosomal recessive, X-linked, and mitochondrial maternal transmission (Meyyazhagan and Orlacchio, 2022) To date, more than 80 genes associated with HSP have been identified (Erfanian Omidvar et al., 2021).

Mutations in the *SPAST* gene are responsible for autosomal dominant HSP type 4 (SPG4), which is the most prevalent subtype, accounting for approximately 15%–40% of all HSP cases in different ethnic backgrounds (Cao et al., 2024; Lu et al., 2018; Alvarez et al., 2010). SPG4 typically manifests as a pure phenotype, although cases of complicated SPG4 have also been documented (Yao et al., 2024). Currently, more than one thousand different variants in the *SPAST* gene have been identified, which include missense and nonsense variants, small deletions, gross deletions, and other types of variants (https://www.hgmd.cf.ac.uk/ac/all.php).

Several studies on HSP or SPG4 have been conducted within the Chinese population across different regions (Cao et al., 2024; Lu et al., 2018; Dong et al., 2018; Wei et al., 2014; Luo et al., 2014); however, the clinical and genetic characteristics of SPG4 specifically in Central China remain unclear. In this study, we aimed to screen for *SPAST* variants in a cohort of 63 HSP families from Central China. Ultimately, we identified 21 *SPAST* variants, including seven novel ones, in 21 of these families. A detailed description of the clinical manifestations and genetic findings were provided, which expanded the clinical and mutational spectrum of SPG4.

## 2 Materials and methods

### 2.1 Patients

From January 2018 to June 2025, we performed a genetic examination of 63 unrelated Chinese patients from Henan province of central China, who were clinically diagnosed with HSP according to the Harding’s criteria (Harding, 1993). All index patients and some family members underwent detailed clinical evaluation. The mode of inheritance was autosomal dominant in 20 families, autosomal recessive in 4 families, and apparently sporadic in 39 cases with no evidence of family history. Among the 63 probands, 21 cases presented with a complicated phenotype. This study was approved by the Ethics Committee of Henan Provincial People’s Hospital. All participants gave their written informed consent.

### 2.2 Genetic analysis

Genomic DNA was extracted from peripheral blood samples from all participants following standard procedures. Whole exome sequencing was performed on the probands using Agilent SureSelect Human All Exon 50-Mb kit (Agilent, Santa Clara, CA, United States) for exome enrichment and the Illumina HiSeq2500 platform (Illumina, San Diego, CA, United States). All identified variants were validated by Sanger sequencing. The average sequencing depth was 136.85× for each sample, and the coverage of the targeted regions was approximate 98.6%. The variants with minor allele frequency (MAF) of < 1% in the Single Nucleotide Polymorphism Database (dbSNP), the Genome Aggregation Database (gnomAD), Exome Aggregation Consortium (ExAC), and the 1000 Genomes Project database (1000G) were kept for further analysis. *In silico* predictions of the functional effect of variants were performed with MutationTaster (https://www.mutationtaster.org), PolyPhen-2 (https://genetics.bwh.harvard.edu/pph2), SIFT (http://sift.jcvi.org), and Human Splicing Finder (http://www.umd.be/HSF). Co-segregation analysis was further performed by Sanger sequencing in the family members. The variants were definited as novel when they were absent in the disease and phenotype databases including Online Mendelian Inheritance in Man (OMIM, http://www.omim.org), ClinVar (http://www.ncbi.nlm.nih.gov/clinvar), the Human Gene Mutation Database (HGMD, http://www.hgmd.org), and Human Phenotype Ontology (HPO, hhtps:hpo.jax.org/app). The novel variants were assigned in accordance with the American College of Medical Genetics and Genomics (ACMG) standards and guidelines (Richards et al., 2015). Copy number variation (CNV) calling from whole exome sequencing was attempted, and exon deletions or duplications were confirmed by multiplex ligation-dependent probe amplification (MLPA).

## 3 Results

**FIGURE 1:**
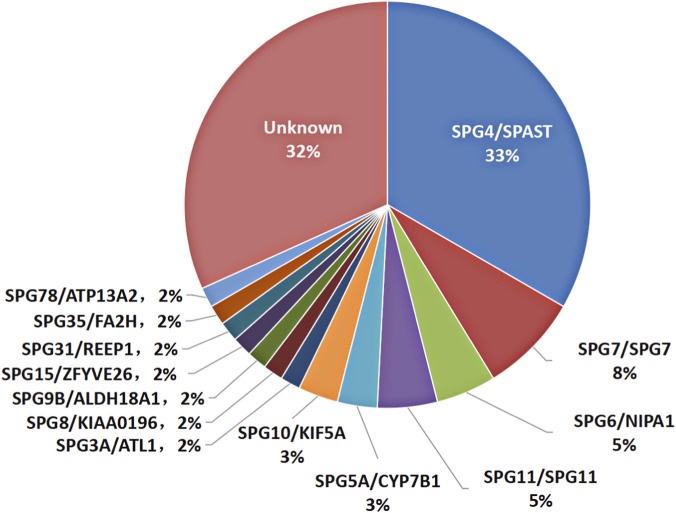
Distribution of genetic cause in 63 HSP families from Central China.

Genetic diagnosis of HSP was established for 43 families with a positive diagnostic yield of 68.3% (43/63). The most frequent subtype was SPG4 (*SPAST*) in 21 probands (21/63, 33.3%), followed by other 12 subtypes including SPG7 (*SPG7*), SPG6 (*NIPA1*), SPG11 (*SPG11*), SPG5A (*CYP7B1*), SPG10 (*KIF5A*), SPG3A (*ATL1*), SPG8 (*KIAA0196*), SPG9B (*ALDH18A1*), SPG15 (*ZFYVE26*), SPG31 (*REEP1*), SPG35 (*FA2H*) and SPG78 (*ATP13A2*) (Figure 1). Four exon deletion variants in *SPAST* were detected by whole exome sequencing in four probands, and confirmed by MLPA.

### 3.1 *SPAST* variants

**FIGURE 2:**
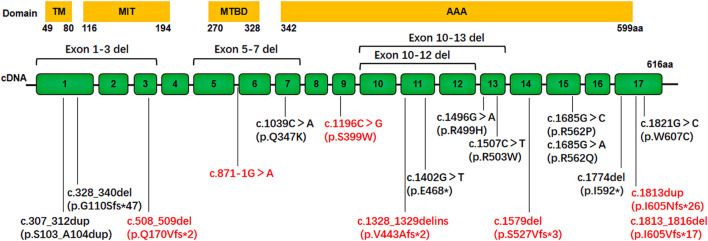
Schematic representation of the mutational location in cDNA of *SPAST* gene detected in our SPG4 cohort (n = 21). The *SPAST* gene contains 17 exons. The novel variants are indicated in red characters.

**FIGURE 3:**
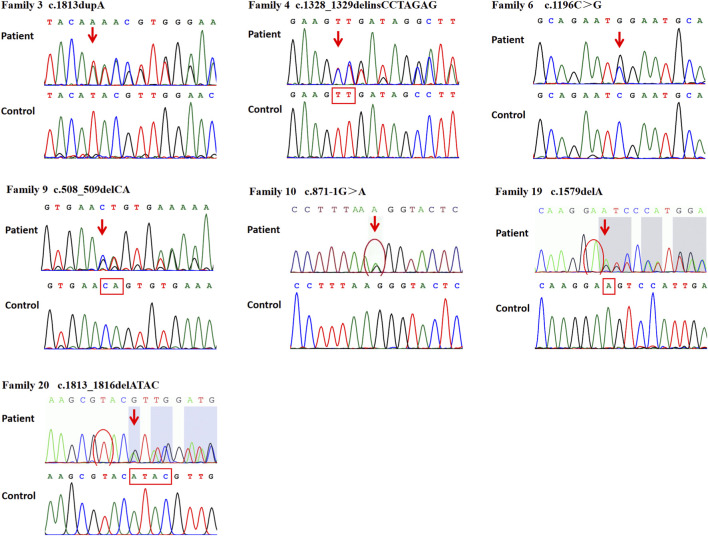
The Sanger sequencing of seven novel variants of *SPAST*.

Twenty-one different *SPAST* variants were identified in 21 probands (Table 1). Among these, 14 variants had been previously reported as pathogenic (Cao et al., 2024; Lu et al., 2018; Dong et al., 2018; Park et al., 2015; Beetz et al., 2006; Crippa et al., 2006; Meyer et al., 2005; Nan et al., 2021; de Bot et al., 2010; Shoukier et al., 2009; McCorquodale et al., 2011; Meijer et al., 2002; Ishiura et al., 2014), while seven were novel. The novel variants included a missense variant, c.1196C>G (p.S399W), three small deletions: c.508_509delCA (p.Q170Vfs*2), c.1579delA (p.S527Vfs*3), and c.1813_1816delATAC (p.I605Vfs*17), one insertion, c.1813dupA (p.I605Nfs*26), one insertion-deletion, c.1328_1329delinsCCTAGAG (p.V443Afs*2), and one splicing variant, c.871-1G>A (Figure 2; Table 2). The Sanger sequencing of seven novel variants was shown in Figure 3.

**TABLE 1:** Clinical and genetic results of 21 probands with SPG4 in the present study.

Index case	Sex	AAO (years)	DD (years)	Inheritance	Hypertonia(UL/LL)	Hyperreflexia(UL/LL)	Hoffmann/Babinski sign	Ankle clonus	Phenotype	Nucleotide change	Amino acide change	Mutation type
1	M	3	35	AD	**−/+**	**−/+**	**−/+**	**+**	P	Exon 5–7 del	​	Deletion
2	M	41	5	AD	**−/+**	**−/+**	**−/+**	**+**	P	c.1774delA	p.I592*	Nonsense
3	M	20	35	AD	**−/+**	**−/+**	**−/+**	**+**	P	c.1813dupA (**#**)	p.I605Nfs*26	Frameshift
4	F	32	4	AD	**−/−**	**+/+**	**+/+**	**+**	P	c.1328_1329delinsCCTAGAG (**#**)	p.V443Afs*2	Frameshift
5	F	31	2	AD	**−/+**	**+/+**	**+/+**	**+**	P	Exon 1–3 del	​	Deletion
6	M	45	4	AD	**−/+**	**−/+**	**−/+**	**-**	P	c.1196C>G (**#**)	p.S399W	Missense
7	M	47	10	AD	**−/+**	**+/+**	**−/+**	**+**	P	c.1821G>C	p.W607C	Missense
8	F	10	20	Sporadic	**−/+**	**−/+**	**−/+**	**-**	C (EP, Dysarthria, Dysphagia)	c.307_312dupTCGGCC	p.S103_A104dup	In-frame duplication
9	M	26	3	Sporadic	**−/+**	**−/+**	**−/+**	**+**	P	c.508_509delCA (**#**)	p.Q170Vfs*2	Frameshift
10	F	49	2	Sporadic	**−/+**	**+/+**	**−/+**	**+**	P	c.871-1G>A (**#**)	​	Splicing
11	M	10	30	Sporadic	**−/+**	**+/+**	**+/+**	**+**	P	c.1496G>A	p.R499H	Missense
12	M	38	1	Sporadic	**−/−**	**−/+**	**−/+**	**+**	P	Exon 10–12 del	​	Deletion
13	M	26	11	Sporadic	**−/+**	**−/+**	**−/+**	**-**	C (EP, CI, VI, Hyposmia)	c.1685G>C	p.R562P	Missense
14	M	36	6	AD	**−/+**	**−/+**	**+/+**	**+**	P	c.1507C>T	p.R503W	Missense
15	M	44	26	AD	**−/+**	**−/+**	**−/+**	**+**	P	c.328_340del	p.G110Sfs*47	Frameshift
16	F	58	8	Sporadic	**−/+**	**−/+**	**−/+**	**+**	P	c.1402G>T	p.E468*	Nonsense
17	M	38	4	Sporadic	**−/+**	**−/+**	**−/+**	**+**	P	c.1039C>A	p.Q347K	Missense
18	M	33	2	Sporadic	**−/+**	**−/+**	**−/+**	**+**	P	c.1685G>A	p.R562Q	Missense
19	M	36	22	AD	**−/+**	**−/+**	**−/+**	**+**	P	c.1579delA (**#**)	p.S527Vfs*3	Frameshift
20	M	35	10	AD	**−/+**	**−/+**	**−/+**	**+**	P	c.1813_1816delATAC (**#**)	p.I605Vfs*17	Frameshift
21	F	55	10	AD	**−/+**	**−/+**	**−/+**	**-**	P	Exon 10–13 del	​	​

AAO, age at onset; AD, autosomal dominant; C, complicated; CI, cognitive impairment; DD, disease duration; EP, epilepsy; F, female; LL, lower limb; M, male; P, pure; UL, upper limb; VI, vision impairment; #, the novel variants; -, negative or absence; +, positive or present.

**TABLE 2:** The seven novel variants of *SPAST* gene identified in our cohort.

Patient	Inheritance	Nucleotide change	Amino acide change	Variant type	ACMG	Evidence
6	AD	c.1196C>G	p.S399W	Missence	Pathogenic	PS4+PM1+PM2+PM5+PP1+PP3
9	Sporadic	c.508_509delCA	p.Q170Vfs*2	Frameshift	Pathogenic	PVS1+PM2+PP3
4	AD	c.1328_1329delinsCCTAGAG	p.V443Afs*2	Frameshift	Pathogenic	PVS1+PM2+PP3
19	AD	c.1579delA	p.S527Vfs*3	Frameshift	Pathogenic	PVS1+PM2+PP3
3	AD	c.1813dupA	p.I605Nfs*26	Frameshift	Likely pathogenic	PM2+PM4+PP1+PP3
20	AD	c.1813_1816delATAC	p.I605Vfs*17	Frameshift	Likely pathogenic	PM2+PM4+PP1+PP3
10	Sporadic	c.871-1G>A	Splicing	Splicing	Pathogenic	PVS1+PM2+PM6+PP3

AD, autosomal dominant; ACMG, american college of medical genetics and genomics.

**FIGURE 4:**
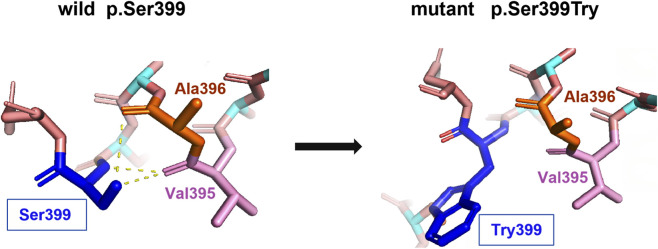
Protein 3D modeling by the SWISS-MODEL software. The amino Ser399 could form polar interactions with Ala396 and Val395; however, the p.S399T variant would result in the loss of this polar interaction.

The novel missense variant c.1196C>G (p.S399W) in exon 9 was found to segregate with the disease in an AD-HSP family. This variant was not found in the ExAC or 1000 Genomes databases. It was predicted to be disease causing by MutationTaster, and probably damaging with a score of 1.000 by PolyPhen-2. The amino acid residue Ser399 is highly evolutionarily conserved across different species, and located within the functional AAA domain (amino acids 342–599). The amino Ser399 could form polar interactions with Ala396 and Val395; however, the p.S399T variant would result in the loss of this polar interaction, as illustrated by the protein 3D modeling using the SWISS-MODEL software (Figure 4). Additionally, a variant at the same position, p.S399L, has been previously reported as pathogenic in several patients (Lu et al., 2018; Wei et al., 2014). According to the standards of ACMG, this novel variant was classified as pathogenic based on evidence PS4+PM1+PM2+PM5+PP1+PP3.

Three novel frameshift variants, p.Q170Vfs*2, p.S527Vfs*3, p.V443Afs*2, were identified in one sporadic patient and in two AD-HSP families, respectively. These variants were predicted to result in truncated spastin proteins, and were classified as pathogenic based on evidence of PVS1, PM2, and PP3. Additionally, two other frameshift variants, p.I605Vfs*17 and p.I605Nfs*26, were identified in two AD-HSP families. These variants were predicted to be translated into spastin proteins that were 4 and 13 amino acids longer than normal, respectively. A similar variant, p.I605Hfs*26, was previously reported in a Korean AD-HSP family (Park et al., 2015). The p.I605Vfs*17 and p.I605Nfs*26 variants were classified as likely pathogenic, supported by evidence of PM2, PM4, PP1, and PP3.

A novel splicing variant, c.871-1G>A, was detected in a sporadic patient. This variant occurred at the critical canonical splice site and was classified as pathogenic, based on evidence of PVS1, PM2, PM6, and PP3.

Four previously reported exon deletions were detected, including exon 1-3, exon 5-7, exon 10–12, and exon 10–13.

Overall, this study revealed the distribution of variant types in the *SPAST* gene: missense variant (7/21, 33.3%), nonsense variant (2/21, 9.5%), splicing variant (1/21, 4.8%),frameshift and in-frame duplication variant (7/21, 33.3%), and exon deletions (4/21, 19.0%) (Figure 2; Table 1).

### 3.2 Clinical manifestations of patients with SPG4

**FIGURE 5:**
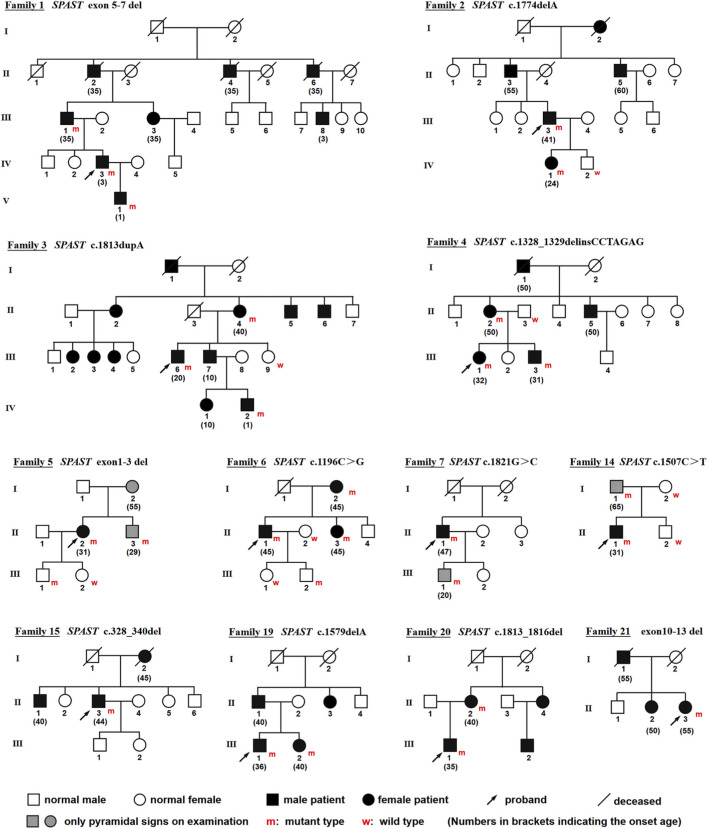
Pedigrees of 12 families with autosomal dominant SPG4. The numbers in the brackets indicate the onset age.

SPG4 was identified in 21 probands, comprising 12 individuals with AD-HSP (12/20, 60.0%) (Figure 5) and 9 sporadic cases (9/39, 23.1%) (Table 1). Male patients were more than females with a ratio of 15:6. All patients presented with gait disturbance due to spasticity or weakness in the lower limbs. Except for one patient (proband 11) who required assistance for ambulation, all other patients maintained the ability to walk independently.

The average age at onset was 34.0 years (ranging from 3 to 58 years), and the mean disease duration was 11.9 years (ranging from 1 to 35 years). In four ADHSP families (family 1–4), the age at onset appeared to be earlier across successive generations (Figure 5). The mean age at onset was 44.3 years (n = 7) in the first generation (family 1 II, family 2 II, family 3 II, and family 4 I), 30.5 years (n = 8) in the second generation, and 16.8 years (n = 6) in the third generation.

As for clinical neurological features, seven probands (33.3%) exhibited lower limb weakness. Pyramidal involvement was noted as indicated by the presence of the Babinski sign and lower limb hyperreflexia both in 21 patients (100%), and lower limb hypertonia in 19 patients (90%), as well as ankle clonus in 17 patients (81%) and the Hoffmann sign in four patients (19%). Additionally, two patients experienced sensory deficits, two suffered from urinary incontinence, and four exhibited pes cavus deformity. Impressively, 19 patients presented with a pure form of HSP, while two patients (9.5%) had a complicated form. Specifically, patient 8 had complications including epilepsy, dysarthria, and dysphagia, while patient 13 had epilepsy, cognitive impairment, visual impairment, and hyposmia.

Besides, several families exhibited intra-familial clinical heterogeneity. Notably, proband 5’s mother and brother, along with the son of proband 7, and the father of proband 14, were identified as asymptomatic carriers, demonstrating only pyramidal involvement on physical examination (Figure 5). Furthermore, proband 3 and his mother exhibited relatively mild symptoms, while his younger brother (family 3 III-7) was severely affected, experiencing an onset age of 10 years, and becoming bedridden by the age of 40. Additionally, both sons of proband 5 and proband 6 were carriers without pyramidal involvement at their younger age.

Brain magnetic resonance imaging (MRI) results were normal for all patients, while spinal MRI revealed mild thoracic spinal cord atrophy in two patients.

## 4 Discussion

In this study, we identified 21 unrelated patients with *SPAST* variants among 63 Chinese families with HSP. This resulted in a mutation frequency of 33.3% for the overall cohort of HSP patients. Specifically, the mutation frequency was 60.0% (12/20) in ADHSP cases, and 23.1% (9/39) in sporadic HSP patients. *SPAST-*related SPG4 was the most common subtype of HSP found in both our cohort and previously reported studies (Cao et al., 2024; Méreaux et al., 2022). Notably, the frequency of *SPAST* variants varied across different regions (Table 3). In the Chinese population, the mutation frequency ranged from 16.2% to 40% (Cao et al., 2024; Lu et al., 2018; Dong et al., 2018; Wei et al., 2014; Luo et al., 2014). The largest previous cohort study in China reported a frequency of 28.9% (78/270). Overall, the estimated *SPAST* mutation frequency in all Chinese HSP patients was 27.5% (175/637). In other Asian populations, the mutation frequency was relatively lower, 18.5% in Japan (Ishiura et al., 2014) and between 18.3% and 26.5% in Korea (Park et al., 2015; Yang et al., 2021). In Europe, the mutation frequency ranged from 9.2% to 30.3%, which is lower than that observed in Chinese patients. A relatively high frequency of approximately 30% was reported in studies from Russia (Kadnikova et al., 2019) and Italy (Nanetti et al., 2012). In contrast, lower frequencies of less than 20% were observed in other European populations (Alvarez et al., 2010; Méreaux et al., 2022; Elert-Dobkowska et al., 2015; Mészárosová et al., 2016; Balicza et al., 2016). The largest cohort study conducted to date from France revealed a mutation frequency of only 9.2% (Méreaux et al., 2022). A study from North America reported a mutation frequency of 27.5%, which is similar to that found in the Chinese population (McCorquodale et al., 2011). The variation in *SPAST* mutation frequency across different populations may be attributed to differences in ethnic backgrounds.

**TABLE 3:** The frequency of SPG4 in different regions and percentage of patients carrying *SPAST* rearrangement variants.

Region	SPG4 in general cohort	SPG4 in AD-HSP	SPG4 in sporadic HSP	Rearrangement variant	Reference
Central China	**33.3% (21/63)**	**60.0% (12/20)**	**23.1% (9/39)**	**19.0% (4/21)**	This study
China	40.0% (22/55)	56.4% (22/39)	​	4.50% (1/22)	Lu et al. (2018)
Southwest China	35.5% (11/31) pHSP	40% (4/10)	33.3% (7/21)	45.5% (5/11)	Wei et al. (2014)
China	28.9% (78/270)	​	​	28.2% (22/78)	Cao et al. (2024)
China	22.5% (27/120)	44.4% (24/54)	4.5% (3/66)	29.7% (8/27)	Luo et al. (2014)
China	16.2% (16/99)	​	​	18.8% (3/16)	Dong et al. (2018)
Korea	26.5% (49/185)	​	​	20.4% (10/49)	Beetz et al. (2006)
Korea	18.3% (19/104)	​	​	5.30% (1/19)	Kadnikova et al. (2019)
Japan	18.5% (32/129)	55.1% (27/49)	7.9% (5/63)	15.6% (5/32)	Méreaux et al. (2022)
Russia	30.3% (37/122)	37.7% (26/69)	20.7% (11/53)	27.0% (10/37)	Nanetti et al. (2012)
Italy	29.2% (19/65)	34.9% (15/43)	21.1% (4/19)	10.5% (2/19)	Elert-Dobkowska et al. (2015)
Poland	18.5% (40/216)	38.8% (33/85)	5.4% (7/131)	37.5% (15/40)	Mészárosová et al. (2016)
Czech	18.3% (48/263) pHSP	​	​	8.30% (4/48)	Balicza et al. (2016)
Hungary	17.2% (10/58)	​	​	​	Rossi et al. (2022)
Spain	14.6% (54/370)	31.2% (44/141)	4.4% (10/229)	3.70% (2/54)	Alvarez et al. (2010)
France	9.2% (142/1550)	​	​	11.3% (16/142)	Yang et al. (2021)
North America	27.5% (33/120)	​	​	3% (1/33)	Meijer et al. (2002)

AD-HSP, autosomal dominant hereditary spastic paraplegia; pHSP, pure hereditary spastic paraplegia.

The bold values indicate the results of our study.

The phenotype of our patients with SPG4 was comparable to that described in other studies (Rossi et al., 2022). The mean age of disease onset typically occurred in the fourth decade, although there was considerable variability in onset age both within and between families. The reported onset age ranged from 0 to 80 years (Rossi et al., 2022). A bimodal distribution of onset age had also been reported, with the first peak occurring in the first decade and the second between the fourth and the fifth decades (de Bot et al., 2010; Rossi et al., 2022; Parodi et al., 2018). However, in our cohort, only one patient experienced onset before the age of 10, which could potentially be due to the relatively small size of our SPG4 cohort. Regarding the age of onset, we also observed anticipation in some families in our cohort, where the onset age was earlier across successive generations. This was consistent with previous reports (Cao et al., 2024; Rossi et al., 2022). The possible explanations for anticipation might be increased disease awareness in younger generations, other modifying genetic factors, or ascertainment bias. The disease generally progressed slowly, as most patients in our cohort were still able to walk independently. Nevertheless, there was noted variability in the severity of spasticity among patients with SPG4 (Yao et al., 2024). Several factors might be involved in the variability, such as epigenetic mechanisms, environmental influences, or modifier genes.

SPG4 is commonly recognized as a typically pure form of HSP. However, an increasing number of cases with complicated phenotype has also been documented. In our cohort, 9.5% of patients exhibited complicated HSP, which was lower than the figures reported in other studies. For instance, two studies from China (Lu et al., 2018; Yao et al., 2024) found rates of 22.2% and 27.3%, respectively. An Italian cohort (Rossi et al., 2022) reported a rate of 26.6%, and a Dutch study (de Bot et al., 2010) presented a rate of 22.4%. The complications associated with complicated HSP included intellectual disability, ataxia, polyneuropathy, dysarthria, epilepsy, distal muscle wasting, vision impairment, hearing impairment, and so on (Lu et al., 2018; Yao et al., 2024; de Bot et al., 2010; Rossi et al., 2022). Notably, the hyposmia observed in our patient had not been previously reported in SPG4 patients or patients with other SPG subtypes. Hyposmia might be a new complication associated with SPG4, but given that this was a single observation and no literature was found to support this plausibility, due attention should be paid to hyposmia in subsequent observational studies on SPG4 and other SPG subtypes.

The *SPAST* gene is located on chromosome 2p22.3 and contains 17 exons. To date, more than one thousand variants have been identified worldwide. In this study, we identified 21 pathogenic or likely pathogenic variants, including seven novel variants, which expanded the mutational spectrum of *SPAST*. There was no hotspot variant in our cohort as well as in others (Yao et al., 2024; Wei et al., 2014; Rossi et al., 2022). The variant types included 33.3% missense variants, 9.5% nonsense variants, 4.8% splicing variants, 33.3% frameshift or in-frame duplication variants, and 19.0% gross rearrangements. These proportions of variant types were in line with previous reports, in which missense and frameshift variants were the most common types, accounting for 31% and 22%, respectively (Yao et al., 2024). Significantly, as shown in Table 3, rearrangements of *SPAST* were a frequent cause of SPG4, accounting for about 10%–30% of all variants. Most rearrangements were exon deletions, while a few were exon duplications (Cao et al., 2024; Dong et al., 2018; Rossi et al., 2022). Therefore, it was critical to use MLPA to detect rearrangement variants in SPG4. It had been previously reported that patients harboring deletion variants of *SPAST* had earlier onset age than those with point variants (Depienne et al., 2007), while, it was not always observed in our patients or in other cohorts (Cao et al., 2024). Among the 17 point variants in *SPAST* in our cohort, 58.5% (10/17) were located in the AAA (*A*TPases *a*ssociated with diverse cellular *a*ctivities) cassette domain, indicating it as a hot mutated region of *SPAST* (Yao et al., 2024; Dong et al., 2018) (Figure 2).

This study has several limitations. Fisrt, we used whole exome sequencing rather than whole genome sequencing. Therefore, complex structural variants, deep intronic variants, or distal regulatory variants may have been missed. Second, reanalysis of whole exome sequencing data was not performed. These may be explainations for those genetically unresolved families. Future studies integrating whole genome sequencing, reanalysis of whole exome sequencing data, and evaluation of novel HSP-related genes may further increase the diagnostic yield.

In conclusion, we identified 21 families with SPG4 in a cohort of 63 HSP families from Central China. The mutation frequency of the *SPAST* gene was 33.3% in our HSP cohort. Anticipation and complicated phenotype can be observed in SPG4 patients. The hyposmia might be a new phenotype associated with SPG4. *SPAST* rearrangement is a common cause of SPG4, and should be considered during genetic analysis. The novel *SPAST* variants identified in this study expand the mutational spectrum of SPG4.

## Data Availability

The data presented in the study are publicly available. The name of the repository and accession numbers can be found at: https://www.ncbi.nlm.nih.gov/clinvar/advanced, SCV006309143, SCV006309144, SCV006309145, SCV006309146, SCV006309147, SCV006309148, SCV006307953.
